# Association between clinical oral health status and perceived oral health in different age groups

**DOI:** 10.7717/peerj.14152

**Published:** 2022-10-03

**Authors:** Ayesha Fahim, Rizwan Mahmood, Irsam Haider, Mamoona Luqman, Ifra Ikhlaq, Tariq Mahmood, Mohammad Khursheed Alam

**Affiliations:** 1 University College of Dentistry, University of Lahore, Lahore, Pakistan; 2 School of Dental Sciences, Universiti Sains Malaysia, Kota Bharu, Kelantan, Malaysia; 3 Azra Naheed Dental College, Lahore, Pakistan; 4 Lahore Medical and Dental College, Lahore, Pakistan; 5 University of Sharjah, Sharjah, United Arab Emirates; 6 College of Dentistry, Jouf university, Al-Jouf, Saudi Arabia; 7 Center for Transdisciplinary Research (CFTR), Saveetha Dental College, Saveetha University, Chennai, India; 8 Faculty of Allied Health Sciences, Daffodil International University, Dhaka, Bangladesh

**Keywords:** Self-assessment, Epidemiology, Oral cavity, Global health

## Abstract

**Objective:**

The perceived oral health refers to the very own perception of a person’s oral health (OH). This study aims to explore the association of perceived oral health status (PSR-OHS) with clinically determined OHS in three age groups: young adults, adults and older adults. This study also aims to identify demographic, socio-economic and/or clinical factors that influence PSR-OHS.

**Methods:**

A cross-sectional study was conducted in ten different dental hospitals of Pakistan. The one-way ANOVA test was used to analyze patient’s demographic distribution with PSR-OHS and oral functions. The complex sample general linear model was used to determine association between clinical OH and PSR-OHS. Analyses of each age group were conducted separately.

**Results:**

A total of 1,804 outdoor patients participated in the study, out of which 660 were young adults, 685 adults and 459 were older adults. Overall self-perception of all age groups about their oral health was ‘good’ (mean = 3.71). Female gender and education status were a significant factor in young adults and adults. Family income affected PSR-OHS of only the adult age group. Frequent visit to dental clinic and preventive reason of dental attendance were associated with good PSR-OHS. DMFT score, prosthesis score and periodontal score also affected the PSR-OHS of individuals. Association between PSR-OHS and clinical examination was confirmed by complex general linear model.

**Conclusion:**

There are differences in the perceived oral health status of young adults, adults and older adults. The variables, age, education, family income, DMFT score, prosthesis score and periodontal score directly influence the self-perception of individuals.

## Introduction

Health literacy (HL) is defined as the ability of individuals to perceive and understand the potential health risks, and make appropriate decisions to manage the disease, subsequently becoming autonomous in their health choices (Batista, Lawrence & Sousa, 2017). HL is now viewed as a critical element in the healthcare system and holds paramount significance in public health agendas (Kanupuru, Fareed & Sudhir, 2015; Kickbusch et al., 2017). Strong evidence exists that links HL to treatment outcomes, compliance to treatment regimens and mortality ratio (Berkman et al., 2011). Just recently, a paradigm shift occurred when oral health literacy (OHL) caught attention of the scientific community. OHL is the degree to which individuals understand basic oral health requirements and take suitable decisions to correct problems, if any (Firmino et al., 2018).

Self-perception refers to an ample and coherent pattern of beliefs related to the manner in which an individual sees himself (Miller, Zivnuska & Kacmar, 2019). The phenomena of subjective self-perception not only provides an insight on how patients perceive their health, it also highlights the social, psychological and functional impact of a disease on an individual’s life (Fagundes et al., 2021). The perceived oral health (PSR-OHS) refers to the subjective perception of a person’s oral health (OH). It differs from the objective oral assessment which is conducted by the dentist. The perceived self-assessment of health implies to questions such as “Generally, would you rate your oral health as excellent, good, fair, or poor?” and surveys based on questionnaires in which patients are asked to evaluate their own health parameters. Such tools are frequently used in epidemiological surveys by health workers which enable them to promote health services effectively. Essentially, such practices not only facilitate the need of regular dental checkups but also helps dentists in treatment planning and to accumulate vital information regarding oral health. In general, PSR-OHS is not just limited to clinical aspects like dental caries or periodontitis, but has great impact on factors such as patient to dentist interaction, OHL, and dental neglect (Kotha et al., 2017). Numerous studies indicated that poor oral health was strongly linked to low HL and lesser use of health services (Berkman et al., 2011), and individuals with low HL were likely to miss their dental appointments (Holtzman et al., 2014). Previous studies have stressed the importance of agreement between the objective dental assessment and the subjective self-perception in order to improve and maintain good oral conditions of an individual (Kim et al., 2018).

In Western countries there are oral health policies regarding routine dental checkups. A 6-monthly dental checkup is required by few states in America (Clarkson et al., 2020). Similarly, dental reports are mandatory for admissions or job applications in USA and European countries (Winkelmann et al., 2022). The oral healthcare system in Pakistan differs considerably. Routine dental checkups are still uncommon in this country and a vast majority of the population visits dental clinics only after enduring pain or tooth loss (Akram et al., 2020). The difference in healthcare system could affect the self-perception of individuals. Previous literature about PSR-OHS origins from the Western world and there is a dearth of knowledge regarding factors associated with PSR-OHS in a developing country like Pakistan.

Each stage of life has its own resources and opportunities which can influence health (Larson et al., 2018). People from various backgrounds and ages have dissimilar reactions to their oral health statuses. It may be assumed that different factors influence people of different age. Old age is accompanied with various systematic disorders, their perception and preference towards oral health may differ from young adults. Some studies have shown that older patients have negative perception about their oral health (Wong, Ng & Leung, 2019), whereas other studies reveal older adults to positively exaggerate about their oral health (Blizniuk et al., 2017; Bulgareli et al., 2018). Similarly, many of the adolescents are independent at this stage and face challenges about their health and lifestyle, subsequently affecting their PSR-OHS (Bulgareli et al., 2018). Individual social experiences and biological events may influence PSR-OHS of individuals (Fagundes et al., 2021). Thus, knowing the specific aspects affecting subjective health at each stage of life could aid in health planning and in meeting the specific needs of each age group. In this study, we aimed to explore the association of PSR-OHS with clinically determined OHS in three age groups: adolescents, adults and older adults.

## Materials and Methods

### Research ethics

The study was conducted in accordance with the declaration of Helsinki. This study was approved by the Ethical Review Committee of Azra Naheed Dental College (ANDC/RAC/29/02). The participants were informed about the objectives of the study and verbal consent was obtained prior to administering questionnaire.

### Study design

A cross-sectional study was conducted from January till August 2021, in the dental OPD of ten dental hospitals of Pakistan. Five teams comprising of two dentists and one dental assistant each were sent to public and private dental hospitals in all four provinces of Pakistan *i.e*., Punjab (4), Sindh (2), Baluchistan (1), KPK (2) and the capital city Islamabad (1).

### Study population

**Figure 1:**
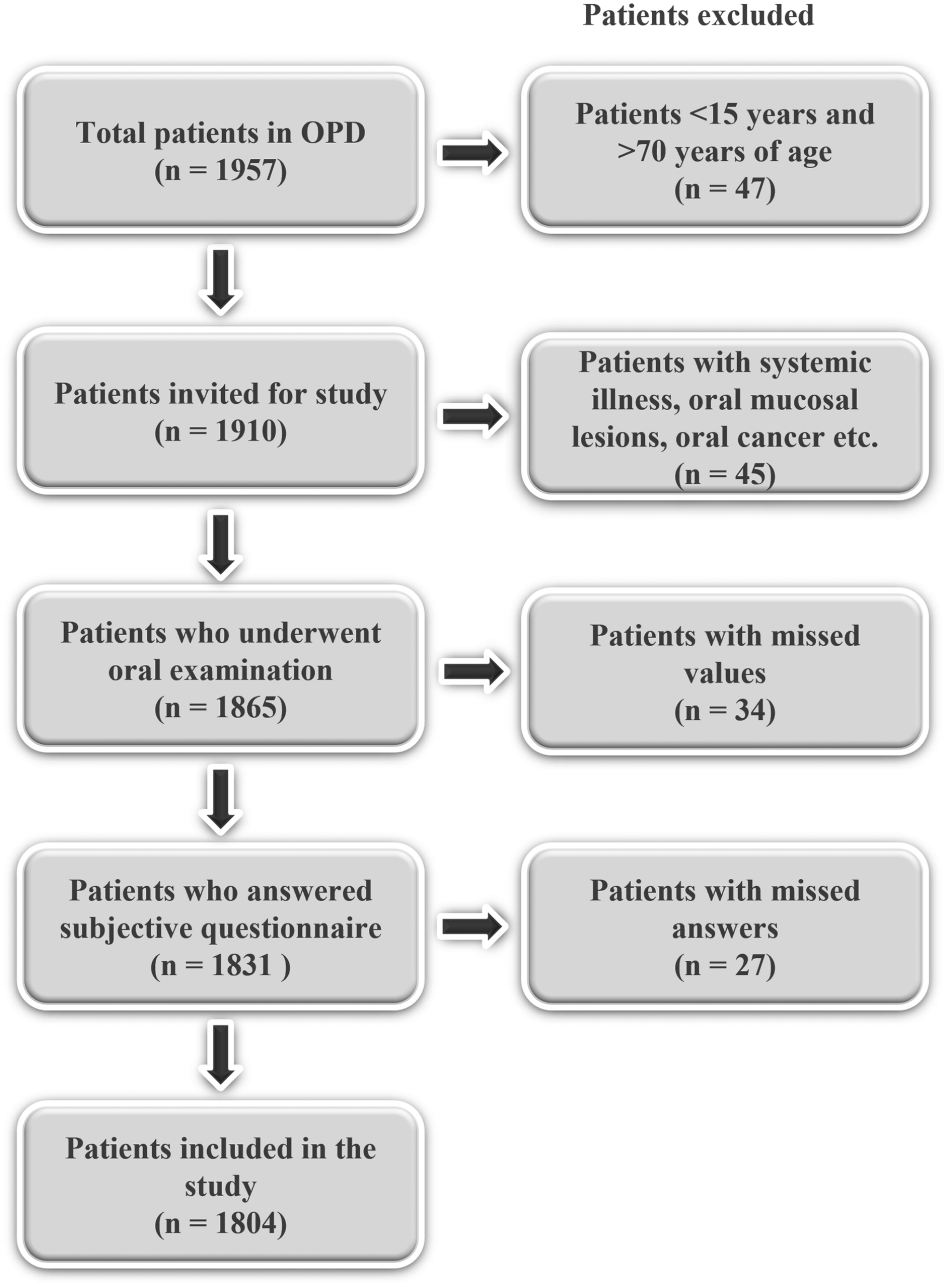
Flowchart showing selection of study participants.

A population-based study was conducted in urban and rural areas of Pakistan. A single stage cluster sampling was done. Each dental team collected data from two hospitals each over the course of 2 months. Thus, the probability of selection was proportional to the population size of Pakistan. During the period of January 2021 to May 2021, a sequential sample of patients was taken. To be eligible for the trial, the participants were required to fall between the age of 15 and 70 and not to be suffering from any underlying systemic diseases including but not limited to mental or physical illness. Participants were divided into three age groups: young adults (15–29 years), adults (30–54 years) and older adults (55–70 years of age). Patients with one or more missing answers were excluded from the study (Fig. 1). Patients with mixed dentition (age < 15) and those with high probability of edentulism (age > 70) were excluded.

### Data collection

Five teams were prepared for data collection. Each team was trained in a 12-h workshop on interviewing and examining the patients to ensure uniformity amongst oral examiners. Training was conducted by a consultant specialist examiner to achieve agreement and overall standardization. The minimum acceptable kappa value for each examiner, age group and condition studied was 0.65. The oral health conditions to be assessed during clinical examination were those recommended by the World Health Organization (WHO, 2013). The PSR-OHS of patients was recorded by trained interviewers using a structured questionnaire.

### Exposure variables

The participants were evaluated based on a structured pre-validated questionnaire comprising of both subjective and objective determinants (Kim et al., 2018). Sociodemographic variables included gender which was recorded as male or female. Data about formal education was calculated as illiterate, primary/secondary or graduation. The family income of patients was dichotomized as either low or high and it was adjusted for family size. It was determined as the gross household income, divided by the square root of the number of household members. According to the OECD scale, low-income families are those whose net income is below 50% of the median income of the entire population (Brandolini & Smeeding, 2012). At the time of data collection, the median income of Pakistani population was Rs. 90,000 per month (available at ceicdata.com), thus individuals with family income of less than Rs. 90,000 per month were considered ‘Low-income families’ and those with family income of more than Rs. 90,000 per month were considered ‘High-income families’ Attendance to dentist was measured by asking about the time of last visit (<1 year or >1 year ago), reason for attending the dentist (preventive or curative) and the type of health facility visited (public or private).

Clinical objective determinants of oral health status were also recorded. Caries status was observed by utilizing the DMFT (Decayed, missing, and filled teeth) index (Moradi et al., 2019). For current study, we split the index into decayed and filled teeth (DFT) and missed teeth (MT), each of which were counted as ≤ or > than the mean. Clinical evaluation was carried out by two dentists after reaching 100% concordance with the calibration process to record DMFT score. Prosthesis score was dichotomized using a six-level scale (non-prosthesis (0), one crown/bridge (1), two or more crowns/bridges (2), only partial dentures (3), bridge and partial dentures (4), and complete denture (5) and was calculated as the sum of observation of upper and lower jaws (Deeb et al., 2020). Thus, the minimum prosthesis score was ‘0’ and maximum prosthesis score was ‘10’. Periodontal index (CPI) was calculated using five-level scale (healthy periodontal status (0), gingival bleeding on probing (1), calculus and bleeding (2), periodontal pocket of 4–5 mm (3), and periodontal pocket >6 mm (4) (Singh et al., 2021).

### Dependent variable

PSR-OHS status of the participants was evaluated based on the following questions: “How do you see your oral health in general?” with the given options excellent (5), good (4), fair (3), poor (2), very poor (1), as conducted in previous studies (Kim et al., 2018) with maximum possible score of 5.

### Statistical analysis

Initially, descriptive analyses were performed. The one-way ANOVA test was used to analyze patient’s demographic distribution with PSR-OHS and oral functions. The two-tailed complex sample general linear model was used to determine association between clinical OH and PSR-OHS. A stepwise approach was carried out using set of variables. In model I, we analyzed DFT score, MT score, prostheses score and CPI score. In model II we added model I and demographic status like age and gender. In model III we added model II and socioeconomic status like education, family income and visit to dentist. The regression line for multivariable regression was analyzed using Y= a + b_1_ × X_1_ + b_2_ × X_2_+ …+ b_n_ × X_n_, where Y = dependent variable, X_i_ = independent variables, a = constant (y-intersect), b_i_= regression coefficient of the variable X_i._ Analyses for each age group were undertaken separately. Level of significance was set at *p* < 0.05. Statistical analyses were performed with the Statistical Package for the Social Sciences (SPSS), version 22 (IBM SPSS Statistics for Windows, Armonk, NY, USA). The multiple regression analyses were done using the Number Cruncher Statistical System (NCSS) version 12 (NCSS, LLC, East Kaysville, UT, USA).

## Results

A total of 990 (54.8%) participants were female and 814 (45.1%) were male. The demographics of study participants in shown in Table 1. Of all the participants, there were 660 young adults, 685 adults and 459 older adults. The frequency and prevalence of poor PSR-OHS is shown in Table 2. There is significant gender difference of PSR-OHS in young adults and adults, where female participants have a higher rate of poor self-perception. However, in older adults, there is no significant gender difference of self-perception. The rate of poor PSR-OHS has decreased with education and there is significant difference between self-perception in all three age groups. The rate of poor PSR-OHS decreases significantly with increased monthly income of adult group. However, there is no significant difference in young adults and older adults. Participants who have attended dental clinics within the last year and those who have visited for preventive purposes have significantly lower rate of poor PSR-OHS in all three age groups. Participants with prevalence of decayed, missed teeth, those in need of prostheses and those with gingivitis had poorer PSR-OHS than their counterparts.

**Table 1:** Demographics of study population.

Variable	Frequency (N) (1804)	Percentage (%)
Socio-demographic measures		
Sex		
Male	814	45.1
Female	990	54.8
Education		
Illiterate	444	24.6
Primary/secondary	843	46.7
Graduation	517	28.6
Family income		
Low	848	47.0
High	956	52.9
Time of last dental attendance		
<1 year	776	43.0
>1 year	1,028	56.9
Type of healthcare service		
Private	789	43.7
Public	1,015	56.3
Reason for attending dental care		
Preventive	416	23.0
Curative	1,388	76.9
Clinical measures		
Decayed, filled Teeth (DFT)		
≤Mean	1,075	77.8
>Mean	729	40.4
Missing teeth (MT)		
≤Mean	1,048	58.1
>Mean	756	41.9
Prostheses score (0–10)		
≤Mean	1,020	56.5
>Mean	784	43.4
Periodontal score (0–4)		
≤Mean	989	54.8
>Mean	815	45.2

**Table 2:** Frequency and prevalence of poor self-perceived oral health according to demographic and socio-economic characteristics dental attendance and dental clinical measures in three age groups.

Variable	Young adults *N* = 660 (36.58%)			Adults *N* = 685 (37.97%)			Older adults *N* = 459 (25.44%)		
	*N*	Prevalence of poor PSR-OHS (95% CI)	*P* value*	*N*	Prevalence of poor PSR-OHS (95% CI)	*P* value*	*N*	Prevalence of poor PSR-OHS (95% CI)	*P* value*
Socio-demographic measures									
Sex									
Male	311 (46.6%)	32.4 [25.4–40.2]	0.012	330 (48.2%)	53.0 [48.7–57.3]	0.02	173 (39.9%)	46.0 [40.5–51.5]	0.142
Female	349 (53.4%)	54.5 [40.0–49.1]		355 (51.8%)	68.6 [59.8–65.4]		286 (60.1%)	44.8 [40.4–49.3]	
Education									
Illiterate	143	54.5 [35.1–46.2]	0.001	76	67.4 [62.9–71.5]	0.031	225	27.4 [62.9–71.5]	0.160
Primary/secondary	371	38.1 [32.3–44.3]		314	53.9 [50.3–57.5]		158	32.8 [45.7–59.8]	
Graduation	146	24.5 [35.1–46.2]		295	27.4 [62.9–71.5]		76	22.7 [39.1–46.3]	
Family income									
Low	274	36.6 [33.2–40.1]	0.086	300	68.4 [50.1–56.7]	0.01	274	45.0 [38.8–45.3]	0.175
High	386	40.0 [35.2–47.0]		385	44.7 [61.0–68.3]		185	47.9 [41.4–54.5]	
Time of last dental attendance									
<1 year	198	37.1 [31.7–42.9]	0.044	325	42.4 [49.3–55.5]	<0.041	253	39.1 [33.0–45.5]	<0.001
>1 year	462	51.5 [34.7–48.6]		360	67.5 [64.0–70.8]		206	58.1 [44.2–52.0]	
Type of healthcare service									
Private	200	51.3 [32.8–42.1]	0.079	330	55.9 [52.7–59.0]	0.166	259	52.3 [38.5–46.3]	0.122
Public	460	50.2 [33.8–46.8]		355	59.2 [59.8–68.3]		199	50.6 [43.3–57.9]	
Reason for attending dental care									
Preventive	179	27.1 [21.4–33.8]	0.003	168	28.7 [34.9–42.5]	0.025	69	38.7 [24.0–34.0]	0.033
Curative	481	56.4 [41.4–51.6]		517	45.6 [62.5–68.6]		390	69.0 [45.3–52.8]	
Clinical measures									
Decayed, filled Teeth (DFT)									
≤Mean	514	32.6 [28.8–36.6]	0.021	231	51.9 [49.0–54.8]	0.036	330	41.4 [37.7–45.3]	0.04
>Mean	146	54.9 [47.0–62.4]		454	79.1 [74.6–82.9]		129	61.9 [55.3–68.0]	
Missing teeth (MT)									
≤Mean	605	37.6 [32.6–42.9]	0.044	382	53.2 [49.6–56.7]	0.042	61	50.9 [44.3–57.3]	0.07
>Mean	55	55.5 [47.6–63.1]		303	70.6 [66.3–74.7]		398	48.2 [38.6–45.9]	
Prostheses score (0–10)									
≤Mean	582	39.2 [34.2–44.3]	0.147	407	57.7 [54.3–60.9]	0.018	31	56.3 [48.7–63.6]	0.027
>Mean	78	39.1 [37.6–89.2]		278	67.6 [62.9–72.1]		428	42.2 [38.6–45.9]	
Periodontal score (0–4)									
≤Mean	460	38.4 [33.4–43.8]	0.004	330	46.0 [41.9–50.1]	0.011	199	44.7 [21.8–27.8]	<0.05
>Mean	200	55.7 [48.3–62.8]		355	71.0 [67.7–74.2]		259	56.0 [51.1–60.7]	

**Notes:**

* By chi-squared test using complex samples crosstabs.

Abbreviations: 95% CI (95% confidence interval), PSR-OHS (perceived oral health status).

The cumulative score of PSR-OHS questionnaire was calculated for three age groups. Overall self-perception of all age groups about their oral health was ‘good’ (mean = 3.71). The self-perceived score of young adults was highest, followed by older adults and adults (*p* = 0.03) (Table 3).

**Table 3:** The mean score of PSR-OHS among three age groups.

Age group	Perceived oral hygiene score (PSR-OHS)	*P* value*
	Mean ± SD	
Young adults (15–29 years)	4.42 ± 2.14	<0.001
Adults (30–54 years)	3.01 ± 1.18	
Older adults (55–70 years)	3.71 ± 3.45	

**Note:**

* By F-test.

The complex sample general linear model was used to determine variables associated with PSR-OHS. The variables DFT, MT, prostheses status and CPI status were significantly associated with PSR-OHS in model I (R^2^ = 0.138). Increase in DFT, MT, prostheses status score and CPI score affected the study participants to have negative or poor self-perception about their oral health. In model II, it was evident that all variables of model I in addition to age were significantly associated with PSR-OHS (R^2^ = 0.129). It was observed that increased age was associated with negative or poor self-perception. Consequently, when model III was adjusted, it was observed that low education and low family income was associated significantly with poor PSR-OHS (R^2^ = 0.075) (Table 4). Based on the estimate coefficient, prosthetic status had the strongest association with PSR-OHS followed by periodontal status, MT score and DFT score in that order. The PSR-OHS score was associated with clinically determined oral hygiene in both non-adjusted and adjusted models.

**Table 4:** The association of demographic, socioeconomic variables, and clinical oral health status with perceived oral health status.

Variable	Self-perceived Oral Health Status (PSR-OHS)								
	Model I (R^2^ = 0.138)			Model II (R^2^ = 0.129)^a^			Model III (R^2^ = 0.075)^b^		
	B	SE	*P* *	B	SE	*P* *	B	SE	*P* *
Age				0.152	0.02	<0.01	0.026	0.003	<0.001
Gender				0.101	0.02	0.05	−0.019	0.063	0.765
Education							0.036	0.035	0.302
Family income							0.101	0.065	0.121
Time of last dental attendance							0.120	0.063	0.055
Type of healthcare							0.032	0.066	0.631
Reason for attending dental clinic							−0.010	0.063	0.122
DFT	0.100	0.02	0.039	0.111	0.02	0.020	0.030	0.02	<0.001
MT	0.421	0.04	0.044	0.444	0.04	0.019	0.280	0.035	<0.001
Prosthesis score	0.779	0.12	0.015	0.759	0.12	0.005	0.636	0.031	<0.001
Periodontal score	0.688	0.06	0.019	0.650	0.06	0.033	0.537	0.025	<0.001

**Notes:**

* By complex samples general linear models.

Age: Young adults = 0, Adult = 1, Older adult = 3. Gender: Male = 0, Female = 1. Education: Illiterate = 0, Primary/secondary school = 1, graduation = 2. Family income: Low = 0, High = 1. Time of last dental attendance: <1 year = 0, >1 year = 1. Type of healthcare: Private = 0, Public = 1. Reason for attending dental clinic: Preventive = 0, Curative = 1. DFT: Mean number of decayed and filled teeth. MT: Mean number of missing teeth. Sum of the prosthesis scores of upper and lower jaws: none = 0, one crown/bridge = 1, two or more crowns/bridges = 2, only partial dentures = 3, bridge and partial dentures = 4, complete dentures = 5. Mean community periodontal index (CPI) of examined sextants at upper and lower jaw; CPI of sextant: healthy = 0, bleeding = 1, calculus = 2, shallow pocket = 3, deep pocket = 4. a, b Model II, Model III include self-perceived oral health status adjusted by demographic variables, respectively.

## Discussion

To our knowledge, this is the first study to identifies the factors affecting self-perceived oral health status in different age groups in Pakistan. This study also explores the association between perceived oral health and clinical oral health. Regarding gender, the female adolescent and adult population had more negative PSR-OHS than the male population, whereas there was no significant difference between the two genders in clinical oral examination. Similar results have been observed in previous studies (Gabardo et al., 2015; Melo et al., 2016; Silva & Oliveira, 2018). Previous studies have hypothesized that social, cultural and political aspects might influence gender response (Gabardo et al., 2015). Females in this society are culturally taught to be more nurturing. This inherent nature might influence them to be more perfectionist about their health and general wellbeing. This could also explain why there was no significant difference between self-perception of older adult female and male participants. Older population tends to have a more realistic approach towards their health (Wong, Ng & Leung, 2019), however, not enough research is available to support this hypothesis.

The level of education was a significant factor for adolescent and adult age groups. PSR-OHS becomes more positive with higher education. This has been shown in several previous studies (Ghaffari et al., 2018; Ismail et al., 2018; Xu et al., 2020). Education enhances oral health awareness and a sense of caution to individuals thus encouraging them to maintain oral health (Levine & Stillman-Lowe, 2019). This, however, was not the case for older adults. The reason could be explained by the fact that this era of the older adults used to reside in urban areas where they cleaned their teeth with ‘miswaak’, a natural tree product. They have a unique sense to care about their oral health by natural ingredients.

While low family income led to a poorer PSR-OHS in each age group, it was an essential factor for only the adult group. In Pakistan, the adult age group is the one with most social responsibilities. Young adults in Pakistan have the lowest earning rate (Mohsin & Syed, 2020). In contrast to Western society, young male adults do not start working until they have graduated (Anum, 2021). More than 85% females do not work right after graduation, mostly owing to social pressures of marriage (Mohsin & Syed, 2020). Young adults are not financially burdened. Similarly, the retirement age in Pakistan is 60 in the public sector (Ashiq & Asad, 2017). Thus, the majority of older adults are not a working class since they rely on the family support system. It has been researched that older adult age is associated with psychological wellbeing and have a better social and financial support than the adult age group (Wright & Brown, 2017). The blend of organizational, individual and social factors put the pressure of earning unto the adult group. Adults also tend to adapt more deleterious habits like smoking tobacco or consuming smokeless tobacco (paan or gutka) more than the other age groups (Naz et al., 2018). Thus, it may be hypothesized that income becomes a great factor in the self-perception of adults. The demographic and socio-economic factors influencing the self-perception of oral health of three age groups in this study highlight how PSR-OHS affect the inequities of this society.

With only few exceptions and in line with previous studies, all clinical examination factors observed in this study, influence the PSR-OHS of individuals of all age groups. The DMFT score affected all three age groups significantly as shown in previous studies (Buldur & Güvendi, 2020; Kaewkamnerdpong & Krisdapong, 2018). It is not mandatory for the public to get a dental checkup every 6 months in Pakistan. The first sign of bad self-oral hygiene that people notice is the presence of caries in mouth. It is then, that individuals feel cautious and look for dental care professional. We purposefully made MT a separate group because this is not a concerning factor in older individuals who have experienced missing teeth and consider this a normal effect of ageing as indicated by previous studies (Banerjee et al., 2018; Spanemberg et al., 2019). The presence of oral diseases seems to be an essential factor in adolescent and adult age groups more than the older individuals. The reason for this may be the fact that older individuals face more systematic diseases than younger adults, thus shifting their main concern towards other health problems rather than oral health. It will be important to monitor future older adults to observe changes in their self-perception since current adults have a different background and oral health concerns.

Regular attendance to dental clinics and attendance due to preventive reasons had a significant impact on good PSR-OHS in all age groups. As mentioned earlier, it is not a mandatory requirement for individuals to visit dental clinics for routine checkups, thus, majority of the people don’t visit often and when they do, it’s almost never for preventive reasons. The results of this study highlight the importance of routine preventive checkups to establish awareness about oral health amongst individuals. The increase in routine dental checkups can directly influence the rate of oral diseases in this population and hence perceived oral health.

The strength of this study is the good population size, meaning that our findings are generalizable for the studied population. Further, the use of a subjective measure as our outcome allowed a more comprehensive interpretation of oral health. The multi-level analyses of contextual factors allowed us to determine the influence of different variables on the primary outcome of this study. There are several limitations in our study. Although we considered multiple factors, we could not analyze all demographic, socioeconomic, general health, and nutritional variables that are related to subjective, and objective OHS. Future studies would also benefit from longitudinal designs to verify the influences on self-perceived oral health over time. Future qualitative studies could be done to determine the reason behind individual’s self-perception of oral health status.

## Conclusions

The results of this study show there are differences in the perceived oral health status of young adults, adults and older adults. The variables, age, education, family income, DMFT score, prosthesis score and periodontal score directly influence the self-perception of individuals.

## Supplemental Information

10.7717/peerj.14152/supp-1 Supplemental Information 1 Values of "Perceived oral health status" of participants and the scores of all variables.

10.7717/peerj.14152/supp-2 Supplemental Information 2 Empty copy of the questionnaire.

10.7717/peerj.14152/supp-3 Supplemental Information 3 Multiple Comparison test for mean difference of PSR-OHS score between age groups.

## Additional Information and Declarations

### Data Availability

The following information was supplied regarding data availability: The raw data is available in the Supplemental Files.
