# Supplementary material for: Association between clinical oral health status and perceived oral health in different age groups
Source: PeerJ. 2022 Oct 3;10:e14152. doi: 10.7717/peerj.14152 (PMC9536301; doi:10.7717/peerj.14152)
Supplement: Supplemental Information 2 [file peerj-10-14152-s002.pdf]

Association between clinical oral health status (OHS) and perceived oral health (PSR-OHS) in different age groups

This questionnaire is designed to be filled by trained dental examiners. The aim of this study is to explore the association of PSR-OHS with clinically determined OHS in three age groups: adolescents, adults and older adults. Participation in the study is purely voluntary. Dental examiners will ONLY collect data of the participants who give verbal consent. Participants have the right to discontinue at their will. The names and other sensitive information of the participants will not be included in data.

Sociodemographic information

Age of participant: _________

Gender: ☐ Male ☐ Female

Education: ☐ Illiterate ☐ Primary / Secondary school ☐ Graduation

Family income: ☐ Low ☐ High

Other information

Attendance to dentist: ☐ < 1 year ☐ > 1 year

Reason for attending the dentist: ☐ Preventive ☐ Curative

Clinical Observation

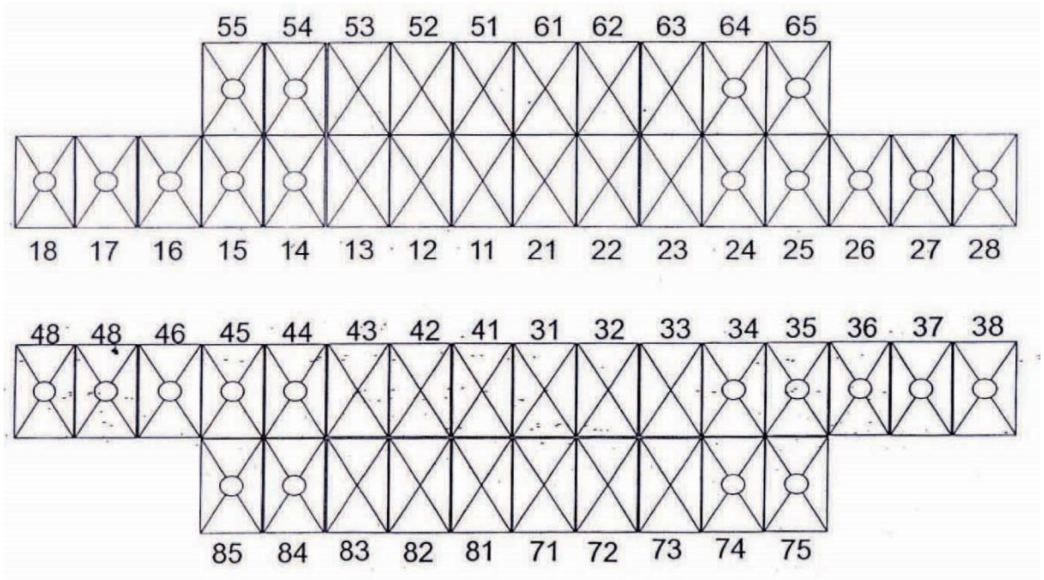

DFT score: _______________

MT score: ________________

Prosthesis score: ___________

0 = one crown / bridge, **1** = two or more crowns / bridges, **2** = only partial dentures, **3** = bridge and partial dentures, **4** = bridge and partial dentures and complete dentures, **5** = bridge and partial dentures and complete dentures and was calculated as the sum of observation of upper and lower jaws

CPI Score: ________________

0 = gingival bleeding on probing, **1** = calculus and bleeding, **2** = periodontal pocket of 4–5 mm, **3** = and periodontal pocket >6 mm

Perceived Oral Health

How do you consider your oral health in general?

☐ Excellent ☐ Good ☐ Fair ☐ Poor ☐ Very poor

Name of dental examiner: __________ Date: __________

Signature of dental examiner: __________
