# Supplementary material for: Association between clinical oral health status and perceived oral health in different age groups
Source: PeerJ. 2022 Oct 3;10:e14152. doi: 10.7717/peerj.14152 (PMC9536301; doi:10.7717/peerj.14152)
Supplement: Supplemental Information 3 [file peerj-10-14152-s003.docx]

**Table 3.1 Multiple Comparison test for mean difference of PSR-OHS score between age groups**

| I | J | I-J |
| --- | --- | --- |
| Young adults (15-29 years) | Adults (30-54 years) | 1.41 |
|  | Older adults (55-70 years) | 0.71 |
| Adults (30-54 years) | Young adults (15-29 years) | -1.41 |
|  | Older adults (55-70 years) | -0.7 |
| Older adults (55-70 years) | Young adults (15-29 years) | -0.71 |
|  | Adults (30-54 years) | 0.70 |
